# Lethal *Pneumocystis jiroveci *pneumonia 24 Years After Kidney Transplantation

**DOI:** 10.5812/numonthly.13605

**Published:** 2014-03-09

**Authors:** Babak Rezavand, Mohammad Javad Hosseini, Morteza Izadi, Abbas Mahmoodzadeh Poornaki, Javid Sadraei, Behzad Einollahi, Mohammad Reza Rezaimanesh, Ozra Bagheri, Jahangir Abdi

**Affiliations:** 1 Department of Parasitology, School of Medicine, Zanjan University of Medical Sciences, Zanjan, IR Iran; 2 Molecular Biology Research Center, Baqiyatallah University of Medical Sciences, Tehran, IR Iran; 3 Health Research Center, Baqiyatallah University of Medical Sciences, Tehran, IR Iran; 4 Department of Parasitology, Medical School, Tarbiat Modares University, Tehran, IR Iran; 5 Nephrology and Urology Research Center, Baqiyatallah University of Medical Sciences, Tehran, IR Iran; 6 Departmanet of Laboratory Sciences, Health Research Center, Birjand University of Medical Sciences, Birjand, IR Iran; 7 Department of Parasitology, School of Medicine, Ilam University of Medical Sciences, Ilam, IR Iran

**Keywords:** Kidney Transplantation, *Pneumonia, Pneumocystis jiroveci*, Polymerase Chain Reaction

## Abstract

**Introduction:**

*Pneumocystis jiroveci* is an opportunistic infectious fungus in immunosuppressed patients, particularly in ones with acquired immunodeficiency syndrome (AIDS). The use of immunosuppressive drugs especially corticosteroids predisposes the transplanted patients to a variety of infectious diseases including *Pneumocystis *infection. In many developed countries, the incidence of *Pneumocystis jiroveci* pneumonia (PJP) is dwindling in transplant patients receiving appropriate prophylaxis. In this study, definitive diagnosis of *Pneumocystis* infection in a patient receiving kidney transplant was presented.

**Case Presentation:**

The patient was a 45-year-old man with a history of kidney transplantation 24 years ago, admitted to a specialized hospital in Tehran because of fever and respiratory distress. Upon admission, the patient showed symptoms of unconsciousness and shortness of breath. Paraclinical tests and complementary examinations such as microscopic observation and molecular analysis confirmed the definitive diagnosis of *Pneumocystis* infection. Specific treatment with trimethoprim/sulfamethoxazole was carried out alongside other therapeutic measures; but unfortunately the patient did not respond to the specific treatment and died in the course of a progressive disease.

**Discussion:**

The disease progress in these patients can still be fast and deadly. Applying rapid molecular diagnostic techniques to start appropriate and timely treatment is essential. Utilization of such diagnostic methods is recommended in our country.

## 1. Introduction

*Pneumocystis jiroveci* pneumonia (PJP) is a fatal disease in patients with AIDS and those receiving organ transplantations. The use of immunosuppressive drugs, especially steroids, makes transplant patients prone to various infectious diseases (1). *Pneumocystis jiroveci* is attached to type 1 pneumocytes of the lung. This causes a deficiency in oxygen exchange resulting in progressive shortness of breath and death if left untreated (2). In many developed countries, the incidence of PJP in immunosuppressed patients receiving appropriate prophylaxis is declining (3). Herein, we discuss a diagnostic report of fatal *Pneumocystis* in a 45-year-old patient receiving kidney transplant 24 years ago.

## 2. Case Presentation

**Figure 1.**
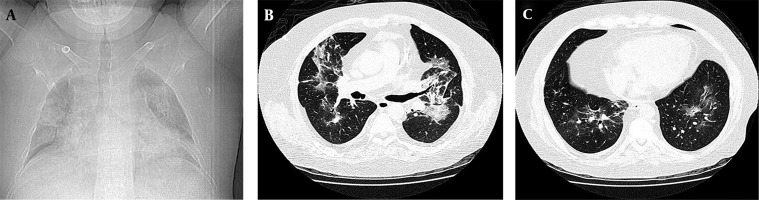
Chest X-ray and CT Section Images Results A. CXR of diffuse bilateral perihilar opacity with extension to periphery of upper and lower lobes; B, C. CT section images, bilateral perihilar alveolar ground glass opacity with extension to periphery of the upper and lower lobes.

**Figure 2.**
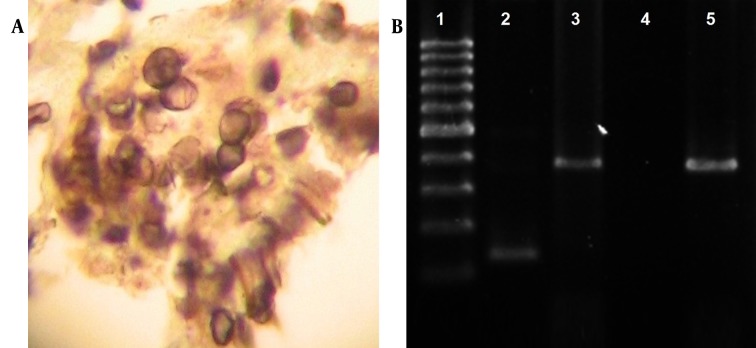
A. Pneumocystis cysts Stained With GMS Image Magnification 1000x; B. Molecular Amplification of Pneumocystis jiroveci Lane 1, 100 bp ladder; Lane 2, Pneumocystis jiroveci nested PCR result using PAZ102-E, pAZ102 and L2 primers; Lane 3, primary Pneumocystis genome amplification using PAZ102-H and PAZ102-E primers; Lane 4, negative control; Lane 5, positive control.

The 45-year-old patient was admitted in a specialized hospital in Tehran with loss of consciousness and symptoms of apnea due to hypoxia. Upon admission, the patient suffered from edema of the hands and legs accompanied with ataxia and low-grade fever. In physical examination, patient was tachypneic. In lung auscultation, fine crackles were remarkable especially in bases of both lungs. In initial examination, the patient’s spouse stated a history of cough and apnea from 10 days ago, aggravated in the last week. The coughs were dry and nonprovocative, and mixed with sputum and blood two days before admission to the hospital. The patient was cytomegalovirus (CMV)-positive with a history of kidney transplant 24 years ago, and had not received any prophylaxis against opportunistic infections. Furthermore, the medical record of the patient indicated a history of receiving three immunosuppressive drugs including cyclosporine, azathioprine and prednisolone after renal transplantation. Chest X-ray (CXR) showed diffuse bilateral perihilar opacity with extension to periphery of upper and lower lobes (Figure 1A). CT section images showed bilateral perihilar alveolar ground glass opacity with extension to periphery of upper and lower lobes. No evidence of cavitation, pleural effusion, abscess formation or adenopathy was observed (Figures 1B and 1C). In further studies, ultrasound of the body showed tense ascites in the abdominal region and an echogenic nodule 17 mm in diameter in the left lobe of the liver. In the laboratory setting, white blood cell (WBCs) and red blood cells (RBCs) counts were reported as 8.2 thous/cumm and 4.15 mL/cumm, respectively. The polymorphonuclear (PMN) cells count was remarkably increased (≈96%). Serum level of sodium and potassium was measured as 136 mEq/L and 5.4 mEq/L respectively, in which the serum level of potassium was higher than the standard values. The values of lactate dehydrogenase (LDH), C-reactive protein (CRP) and erythrocyte sedimentation rate (ESR) are presented in the Table 1. All three reported values were greater than standard. On the fifth day of hospitalization, the patient developed worsening shortness of breath and inability to lie down, and the arterial O_2_ was 69%. The patient was transferred to the intensive care unit; intubation was performed and the patient was connected to mechanical ventilation (MV). The lung lavage sample was positive for *Pneumocystis jiroveci* using gomori methenamine silver staining (Figure 2A). 

**Table 1.** LDH, CRP and ESR Values ^a^

	Results
**LDH, IU/L**	1109
**CRP, mg/L**	59.9
**ESR, mm/h**	16

^a^ Abbreviations: CRP, C-reactive protein; ESR, erythrocyte sedimentation rate; LDH, lactate dehydrogenase.

### 3.1. Molecular Laboratory

Genome of bronchoalveolar lavage (BAL) samples obtained from the patient was extracted using Rima ® Zol FlexiGen kit manufactured by Teifara Company, Iran. The search for specific genome of *Pneumocystis jiroveci* was conducted based on replication of mtLSUrRNA gene. The initial amplification was performed using the initial primers pAZ-102-E and pAZ102-H. Nested polychain reaction (nested-PCR) was performed using the PCR product of the primary phase along with the internal primers pAZ-102-E and pAZ102-L2 (4). The presence of amplified genome was studied after electrophoresis in 1.5% agarose gel stained with ethidium bromide. In this study the positive control sample of *Pneumocystis Jiroveci *isolated and sequenced from human immunodeficiency virus (HIV)-positive patients in Iran with the code number JF733748,which is listed on the World Gene Bank, was used (5).

The *Pneumocystis jiroveci* genome appeared as a 346 bp specific band in the initial amplification, and as a 120 bp band in nested PCR (Figure 2B). After 16 days of hospitalization in the intensive care unit, the patient did not respond to specific treatment with trimethoprim/sulfamethoxazole regarding Centers for Disease Control and Prevention (CDC) standards, and died because of hypoxia and pneumothorax.

## 3. Discussion

To date, the key principle in kidney transplantation has been suppression of allograft rejection. Hence, development of immunosuppressive agents is crucial for successful allograft function. To achieve an intense immunosuppression in the initial days after transplantation, immunosuppressive molecules are used for depleting lymphocytes, diverting lymphocyte traffic, or blocking lymphocyte response pathways aiding maintenance, and reversal of the established rejection (6). One of the inevitable effects of immunosuppressive drugs is undesired consequence of immunodeficiency. Immunodeficiency leads to characteristic infections (such as opportunistic infections) and cancers (7). With increasing the number of immunosuppressed patients in different communities, the importance of opportunistic infectious organisms has become highlighted for clinicians over time. PJP is a result of a dangerous opportunistic organism for patients with acquired immunosuppression, especially AIDS, cancer, and those receiving transplantation (8). The studies showed that the incidence of this disease among kidney transplant patients was 14% (9). Routine laboratory tests provide limited information. Measurement of serum LDH seems to be a useful test that increases during the infection, but this test is nonspecific and serum LDH increases in other conditions such as other pneumonias and lymphoma. Patients with LDH levels two to three times higher than normal have a considerably higher mortality (10). In patients with HIV, the relative risk of PJP is associated with their prophylaxis against *Pneumocystis *infection. If there is no prophylaxis for a patient with pneumonia, risk of recurrence of *Pneumocystis carinii* Pneumonia (PCP) one year after infection would be 70% (11). If trimethoprim/sulfamethoxazole (TMP/SMX) is administered for prophylaxis, the risk of recurrence of PJP will be reduced (12). The above report suggests that despite 24 years of transplant, the risk of opportunistic infections in transplant patients is still inevitable. Therefore, considering the probability of opportunistic infections in these patients is critical. The disease progress in these patients can still be fast and deadly. Applying rapid molecular diagnostic techniques to start appropriate and timely treatment is essential. Utilization of such diagnostic methods is recommended in our country.
