# Corrigendum to: Lethal Pneumocystis jiroveci pneumonia 24 Years After Kidney Transplantation [Published in Nephro Urol Mon. 2014 March; 6(2): e13605. DOI: 10.5812/numonthly.13605]

**DOI:** 10.5812/numonthly.19786

**Published:** 2014-07-05

Here by, authors declare that the corrected authors list and affiliations are:

Babak Rezavand ^1^; Mohammad Javad Hosseini ^2,*^; Morteza Izadi ^3^; Abbas Mahmoodzadeh Poornaki ^1^; Javid Sadraei ^4^; Behzad Einollahi ^5^; Mohammad Reza Rezaimanesh ^6^; Ozra Bagheri ^2^; Jahangir Abdi ^7^

^1^ Department of Parasitology, School of Medicine, Zanjan University of Medical Sciences, Zanjan, IR Iran

^2^ Molecular Biology Research Center, Baqiyatallah University of Medical Sciences, Tehran, IR Iran

^3^ Health Research Center, Baqiyatallah University of Medical Sciences, Tehran, IR Iran

^4^ Department of Parasitology, Medical School, Tarbiat Modares University, Tehran, IR Iran

^5^ Nephrology and Urology Research Center, Baqiyatallah University of Medical Sciences, Tehran, IR Iran

^6^ Department of Laboratory Sciences, Torbat Heydariyeh University of Medical Sciences, Torbat Heydariyeh, IR Iran

^7^ Department of Parasitology, School of Medicine, Ilam University of Medical Sciences, Ilam, IR Iran

Sincerely Yours

Mohammad Reza Rezaimanesh

